# Orexin 2 Receptor Antagonism is Sufficient to Promote NREM and REM Sleep from Mouse to Man

**DOI:** 10.1038/srep27147

**Published:** 2016-06-03

**Authors:** Anthony L. Gotter, Mark S. Forman, Charles M. Harrell, Joanne Stevens, Vladimir Svetnik, Ka Lai Yee, Xiaodong Li, Anthony J. Roecker, Steven V. Fox, Pamela L. Tannenbaum, Susan L. Garson, Inge De Lepeleire, Nicole Calder, Laura Rosen, Arie Struyk, Paul J. Coleman, W. Joseph Herring, John J. Renger, Christopher J. Winrow

**Affiliations:** 1 Department of Neuroscience, Merck & Co. Inc., Kenilworth, NJ, USA; 2 Department of Translational Medicine, Merck & Co. Inc., Kenilworth, NJ, USA; 3 Department of in vivo Pharmacology, Merck & Co. Inc., Kenilworth, NJ, USA; 4 Department of Biostatistics and Research Decision Sciences, Merck & Co. Inc., Kenilworth, NJ, USA; 5 Department of Pharmacokinetics Pharmacodynamics and Drug Metabolism, Merck & Co. Inc., Kenilworth, NJ, USA; 6 Department of Medicinal Chemistry, Merck & Co. Inc., Kenilworth, NJ, USA; 7 Department of Clinical Neuroscience, Merck & Co. Inc., Kenilworth, NJ, USA

## Abstract

Orexin neuropeptides regulate sleep/wake through orexin receptors (OX_1_R, OX_2_R); OX_2_R is the predominant mediator of arousal promotion. The potential for single OX_2_R antagonism to effectively promote sleep has yet to be demonstrated in humans. MK-1064 is an OX_2_R-single antagonist. Preclinically, MK-1064 promotes sleep and increases both rapid eye movement (REM) and non-REM (NREM) sleep in rats at OX_2_R occupancies higher than the range observed for dual orexin receptor antagonists. Similar to dual antagonists, MK-1064 increases NREM and REM sleep in dogs without inducing cataplexy. Two Phase I studies in healthy human subjects evaluated safety, tolerability, pharmacokinetics and sleep-promoting effects of MK-1064, and demonstrated dose-dependent increases in subjective somnolence (via Karolinska Sleepiness Scale and Visual Analogue Scale measures) and sleep (via polysomnography), including increased REM and NREM sleep. Thus, selective OX_2_R antagonism is sufficient to promote REM and NREM sleep across species, similarly to that seen with dual orexin receptor antagonism.

The orexin signaling pathway drives arousal, as demonstrated by genetic and pharmacologic studies. Circadian oscillations in orexin peptides A and B within cerebrospinal fluid correlate with arousal cycles^1^,^2^, and exogenous administration of the neuropeptides is sufficient to drive wakefulness in rodents, particularly during the inactive phase^3^,^4^. A lack of orexin signaling is associated with narcolepsy, a disorder characterized by uncontrollable sleep episodes and hypersomnolence^5^. Disruption of the gene encoding the orexin 2 receptor is causal for canine narcolepsy^6^. Further demonstration of the importance of orexin in arousal came from mice where ablation of the gene for orexin (*Hcrt*) leads to a narcolepsy-like phenotype, including cataplectic behavior^7^; targeted mutagenesis of the *Hcrtr1* and *Hcrtr2* genes encoding orexin receptors 1 and 2 (OX_1_R and OX_2_R) is associated with a similar narcoleptic phenotype^8^.

Several studies have provided preclinical and clinical proof of concept that orexin receptor antagonists could serve as insomnia therapeutics. Multiple compounds selective for both OX_1_R and OX_2_R, so-called dual orexin receptor antagonists (DORAs), have now shown efficacy in preclinical models and in human patients with insomnia. These include almorexant^9^,^10^, SB-649868^11^, and suvorexant (Belsomra^®^), the first OXR antagonist approved as an insomnia treatment^12^,^13^,^14^.

Extensive evidence suggests that DORAs promote rapid eye movement (REM) and non-REM (NREM) sleep similar to that occurring during the normal resting phase. This has been observed across preclinical species^9^,^15^,^16^ as well as in healthy human subjects and patients with insomnia^17^,^18^,^19^. DORA-induced sleep appears to be indistinguishable from naturally occurring sleep in rodents based on sleep architecture analysis^15^ and DORA administration minimally impacts sleep-stage–specific quantitative electroencephalography (qEEG) power in rats^16^ as well as in healthy human subjects and patients with insomnia^20^.

Preclinically, OX_2_R appears to be critical for the arousal-promoting effects of orexin and, conversely, appears to be primarily responsible for the increased propensity for sleep associated with loss of orexin signaling. In dogs, OX_2_R mutations alone are responsible for the narcoleptic phenotype^6^. In mice, targeted mutagenesis of the gene encoding OX_2_R results in hypersomnolence that is not as pervasive as that seen in OX_1/2_R double mutants, while OX_1_R knockout mice exhibit a subtle, barely detectable sleep phenotype^21^. These findings suggest that OX_2_R single antagonists (2-SORAs) may be sufficient to induce sleep. Indeed, the effects of DORAs on sleep are nearly absent in OX_2_R knockouts^22^, indicating that the effects of DORAs are largely mediated through OX_2_R in mice, but the translation of the predominant role of OX_2_R has yet to be demonstrated in humans.

The sleep-promoting differences induced by DORAs and 2-SORAs across species remain controversial. The sleep-inducing efficacy of DORAs occurs at OX_2_R receptor occupancies of 65% to 80% in rodents^1^, but the impact of OX_1_R antagonism is a matter of debate. Results utilizing almorexant, the 2-SORA JNJ-10397049 (~320x OX_2_R selective), and the partial OX_1_R single antagonist (1-SORA; ~64x OX_1_R selective) SB-408124, suggest that OX_1_R antagonism, which is included in the DORA mechanism of action, attenuates the sleep effects of OX_2_R blockade^23^. Morairty *et al*., however, found dual receptor antagonism (by almorexant) to be more effective at promoting sleep compared with the highly selective 2-SORA EMPA (~900x OX_2_R selective), and observed that maximal sleep promotion with almorexant coincided with occupancy of both OXRs^24^. Given the brainstem localization of OX_1_R, particularly in the locus coeruleus, OX_1_R antagonism may attenuate vigilance state boundaries^25^,^26^,^27^, effectively diminishing the threshold for sleep-state transitions. More recently, the 2-SORA JNJ-42847922 (~80-fold binding selectivity for OX_2_R relative to OX_1_R) was shown to promote NREM sleep in rats while increases in REM duration did not reach significance in the 2 hours following treatment. In a single ascending dose study in human subjects, JNJ-42847922 was effective in promoting somnolence, but effects on sleep architecture remain to be determined^28^.

MK-1064 is a novel 2-SORA whose synthesis, discovery, and initial characterization has been recently described^29^. Here, we assessed the *in vitro* properties of MK-1064 and its sleep-promoting efficacy in preclinical species and human subjects to characterize its mechanism of action to evaluate the potential of OX_2_R antagonism as a therapeutic strategy for insomnia.

## Results

### MK-1064 is highly selective for OX_2_R

MK-1064 was evaluated for its ability to inhibit orexin-peptide-A-induced calcium mobilization in CHO cell lines separately expressing OX_1_R and OX_2_R from mice, rats, rabbits, dogs, rhesus monkeys and humans. Assayed concurrently with other 2-SORAs (MK-3697^30^, 2-SORA-18, 2-SORA-7) and the DORA MK-6096, these results provide a comprehensive comparison of MK-1064 to other compounds across receptors and species (Table 1). The potency of MK-1064 for OX_2_R activation was consistent across species, ranging from a K_b_ of 7 nM in mice to 13 nM in rats. It was 208-fold more selective for hOX_2_R compared with hOX_1_R (1872 nM) and was the most potent of the 2-SORAs, with a K_b_ for hOX_2_R of 9 nM. Similar cross-species results were also seen for binding affinity as measured by radio-ligand displacement, with the K_i_ for MK-1064 binding to OX_2_R ranging from 0.51 nM in rabbit to 1.02 nM in mice (Table 2). The K_i_ for binding to hOX_2_R was 0.52 nM, which was 2885-fold more selective compared with that seen for hOX_1_R binding (1500 nM).

Assessment of binding kinetics indicated that MK-1064 associates with hOX_2_R in a therapeutically advantageous manner (Table 2). The T_1/2ON_ of MK-1064 was 21.1 minutes and is similar to that of the 2-SORA, MK-3697, but faster than that of the DORA, MK-6096. The T_1/2OFF_ for MK-1064 was 110 min, which was similar to that of MK-6096 but faster than that of MK-3697. Affinity for hOX_2_R, as expressed in K_d_ in these experiments, was 0.51 nM, consistent with the K_i_ value determined in multiplex experiments (0.52 nM) and substantiating the cross-species comparisons.

### MK-1064 promotes sleep in rodents selectively through OX_2_R

**Figure 1:**
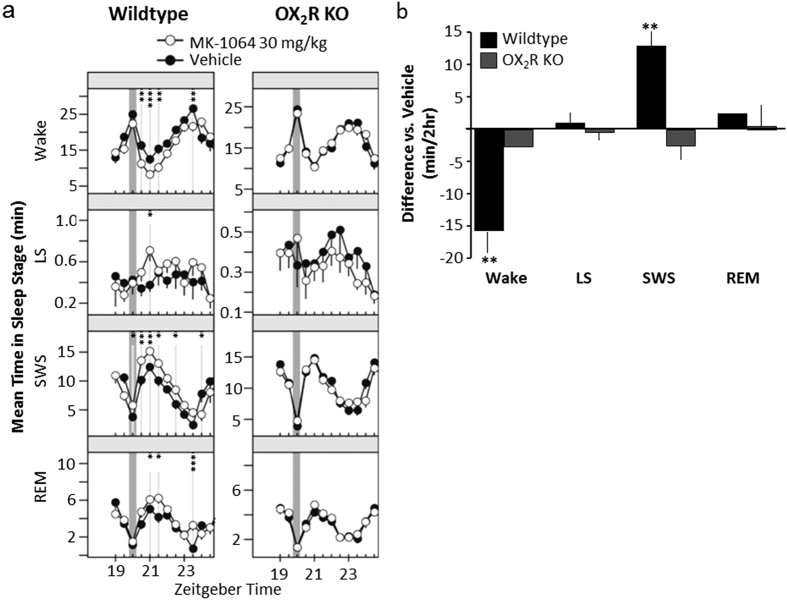
Sleep effects of MK-1064 30 mg/kg are OX_2_R-dependent. (**a**) The mean time spent in active wake, light sleep (LS), SWS, and REM sleep during 30-min intervals was determined in wild-type (left panel; *n* = 5) and OX_2_R knockout (right panel; *n* = 7) mice following administration of MK-1064 30 mg/kg (open circles) relative to vehicle (20% vitamin E TPGS, orally; closed circles) during the late active phase (ZT: 20:00). Time points at which significant differences exist between vehicle and MK-1064 responses are indicated by grey vertical lines (*, **, ***p < 0.05, 0.01, 0.001, respectively; linear mixed-effects model for repeated measures). (**b**) Mean within-subject difference ± standard error of the mean of time spent in active wake, LS, SWS, and REM for 2 hours following oral treatment with MK-1064 (30 mg/kg) versus vehicle (20% vitamin E TPGS) in wild-type and OX_2_R knockout mice is shown (**p < 0.01, one sample t-test for repeated measures compared with vehicle).

Polysomnography (PSG) responses to MK-1064 (30 mg/kg) were evaluated following oral administration during the late active phase (administered at Zeitgeber time [ZT] 20:00) either in wild-type or OX_2_R knockout telemetered mice. In wild-type animals, active wake reduction was accompanied by significant increases in slow-wave sleep (SWS) and REM at time points up to 3.5 hours following treatment (Fig. 1a). Quantitated over 2 hours, these changes in wake and SWS reached significance, whereas REM exhibited a trend toward an increase for wild-type animals (Fig. 1b), similar to that previously observed by others following treatment with other 2-SORAs^23^,^31^. Importantly, all of these changes with MK-1064 were absent in OX_2_R knockout mice (Fig. 1), demonstrating the selectivity of this compound and confirming that OX_2_R antagonism is sufficient to induce sleep.

**Figure 2:**
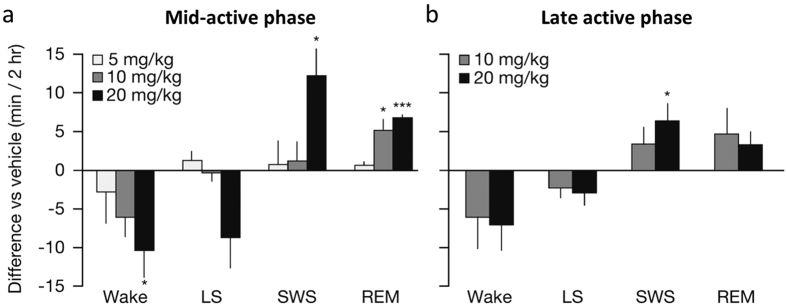
MK-1064 dose-dependently promotes both NREM and REM sleep during the active phase in rats. Mean time in active wake, light sleep (LS), SWS, and REM sleep in adult Sprague-Dawley rats monitored for 2 hours post-dose. MK-1064 (5, 10, or 20 mg/kg) or vehicle (20% vitamin E TPGS, orally) was administered in a balanced crossover design such that each animal received drug and vehicle treatments (7 consecutive days). Treatment occurred during the active phase at ZT 17:00, (**a**) 7 hours prior to lights on (5 mg/kg, *n* = 5; 10 mg/kg, *n* = 7; 20 mg/kg, *n* = 5), or (**b**) just prior to onset of inactive phase (ZT 23:00; 10 mg/kg, *n* = 8; 20 mg/kg, *n* = 7). Mean within-subject change relative to vehicle ± SEM is shown (*, ***p < 0.05 < 0.001, respectively; one sample t-test for repeated measures compared with vehicle).

MK-1064 promoted both REM and NREM sleep in telemetered rats in a time-of-day-dependent manner. When administered during the mid-active phase, MK-1064 at 5, 10 and 20 mg/kg dose dependently attenuated active wake during the 2 hours immediately following compound administration relative to vehicle, with the response to the 20 mg/kg dose reaching statistical significance relative to vehicle (p < 0.05, t-test for repeated measures) (Fig. 2a). Reductions in active wake were accompanied by increases in SWS at the 20 mg/kg dose and increases in REM sleep at both the 10 and 20 mg/kg doses. When administered during the late active phase, 1 hour prior to the inactive period, responses to MK-1064 were less robust, as may be expected in normal rats as orexin levels begin to diminish rapidly during the ensuing light phase^1^. Trends in active wake reduction were accompanied by increases in SWS and REM sleep similar to that seen during the active phase; but, in this case, only SWS reached significance in response to the 20 mg/kg dose (Fig. 2b).

### MK-1064 requires greater OX_2_R occupancies to induce sleep relative to DORA-12

**Figure 3:**
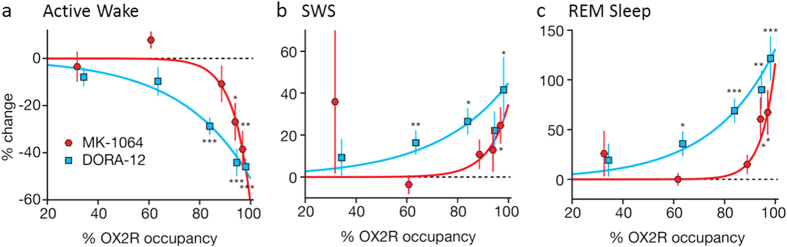
MK-1064 requires higher OX_2_R occupancies to promote sleep relative to DORA-12 in rats. The mean percentage change (±SEM) in (**a**) active wake, (**b**) SWS, and (**c**) REM sleep in response to MK-1064 (at 0.3, 1, 5, 10 and 20 mg/kg) and DORA-12 (at 0.3, 1, 3, 10 and 30 mg/kg) calculated from PSG recordings versus the percentage OX_2_R occupancy determined in rats. Occupancy versus plasma exposure curves were determined and the exposure in satellite animals evaluated in parallel with PSG animals was used to determine corresponding OX_2_R occupancies at C_max_ (1 hour) following treatment. Occupancies at which significant % change in active wake, SWS and REM sleep were observed over the 2 hours following treatment relative to vehicle are indicated (*, **, ***p < 0.05, 0.01, 0.001 respectively; one sample t-test for repeated measures).

OX_2_R occupancy is rate-limiting for sleep promotion by orexin receptor antagonists. DORAs significantly attenuate active wake at OX_2_R occupancies between 65% and 85%^1^,^23^,^24^. Here, we assessed the level of OX_2_R occupancy required by the 2-SORA, MK-1064, to induce changes in active wake, SWS, and REM sleep in rats relative to that seen for DORA-12. As seen in Fig. 3a, significant reductions in active wake, as a percentage of that observed in the 2 hours following vehicle treatment, were observed at exposures achieving 94% and 97% occupancies (unbound [MK-1064]_plasma_ = 317 and 634 μM, respectively), but not at exposures achieving 89% occupancy (unbound [MK-1064]_plasma_ = 159 μM); this indicates that a threshold of 89–94% OX_2_R occupancy is required for significant sleep promotion. In comparison, DORA-12 induces significant active wake changes at lower occupancy levels (83%) in these experiments. Significant SWS changes were induced by MK-1064 at OX_2_R occupancy of 97%, while DORA-12 induced significant SWS at occupancies as low as 63% (Fig. 3b). Similarly, MK-1064–induced changes in REM sleep occurred at 94%, but changes induced by DORA-12 occurred at levels as low as 63% (Fig. 3c). The evaluation of additional DORAs and SORAs revealed a similar overall pattern (not shown). Taken together, these results indicate that higher OX_2_R occupancies are required by 2-SORAs for sleep promotion relative to DORAs and suggest that OX_1_R antagonism by DORAs contributes to sleep-promoting efficacy.

### MK-1064 promotes sleep but not cataplexy in dogs

**Figure 4:**
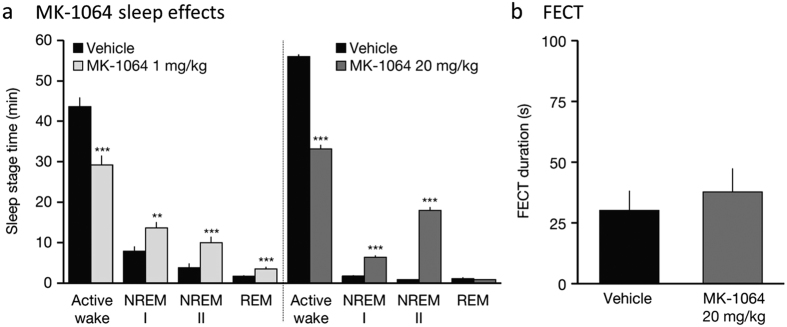
MK-1064 effectively promotes somnolence but not cataplexy in canines. (**a**) Mean time (±SEM) in active wake, NREM I, NREM II, and REM sleep of dogs in the 1 hour following active-phase administration of vehicle (sodium citrate 10 mM, orally) or MK-1064 1 mg/kg (*N* = 6, ZT 03:00) or 20 mg/kg (*N* = 8, ZT 04:00) (linear mixed-effects model for repeated measures ANOVA; **, ***p < 0.05 < 0.001, respectively; t test). (**b**) In coincident analysis, the mean duration (±SEM) to complete FECT following administration of vehicle (sodium citrate 10 mM, orally) or MK-1064 (20 mg/kg) 1 hour after dose administration showed no significant difference between conditions (*N* = 8, paired t-test).

Telemetered dogs exhibited robust sleep promotion following treatment with MK-1064 relative to vehicle (Fig. 4a). Significant active wake reductions were accompanied by increases in NREM I, NREM II, and REM sleep following administration of the 1 mg/kg dose (time at which maximum concentration was reached [T_max_] = 0.3 hours, maximum concentration [C_max_] = 1.0 μM, area under the concentration-time curve [AUC_0–24_] = 3.4 μM*hour). The 20 mg/kg dose (T_max_ = 0.8 hours, C_max_ = 15.3 μM, AUC_0-24_ = 56.5 μM*hour) induced similar changes of greater magnitude in active wake, NREM I and NREM II sleep, the latter of which is characterized by a predominance of SWS or delta sleep. REM sleep was not significantly increased by this higher dose of MK-1064 relative to vehicle in the hour following administration but was increased at several later time points (Supplementary Fig. 1), indicating time and/or exposure dependent effects on NREM and REM sleep.

Genetic data in canines suggest that OX_2_R antagonism may be sufficient to induce cataplexy, as inherited OX_2_R mutations produce a syndrome in dogs resembling human narcolepsy^6^. A standard measure for quantifying narcolepsy with cataplexy in dogs is the food-elicited cataplexy test (FECT). In this paradigm, narcoleptic dogs typically exhibit cataplectic episodes when presented with 10 balls of food arranged in an evenly spaced line and require >50 seconds to complete the FECT, with some animals requiring up to 30 minutes^32^,^33^. Dogs evaluated by FECT 1 hour after a 20 mg/kg administration of MK-1064, a time just after peak plasma levels are reached, showed no cataplectic episodes, and the mean time to complete the FECT was 37.8 seconds (Fig. 4b). This value was not significantly different from vehicle-treated animals, and well below the minimal amount of time typically required for narcoleptic canines to complete the FECT^33^. The MK-1064 dosage used in dogs in these experiments was exceedingly high; plasma concentrations reached 15.3 μM (C_max_), which is 500-fold higher than the minimum needed for sleep-promoting efficacy (0.028 μM)^29^.

### MK-1064 attenuates arousal and promotes NREM and REM sleep in healthy human subjects

**Figure 5:**
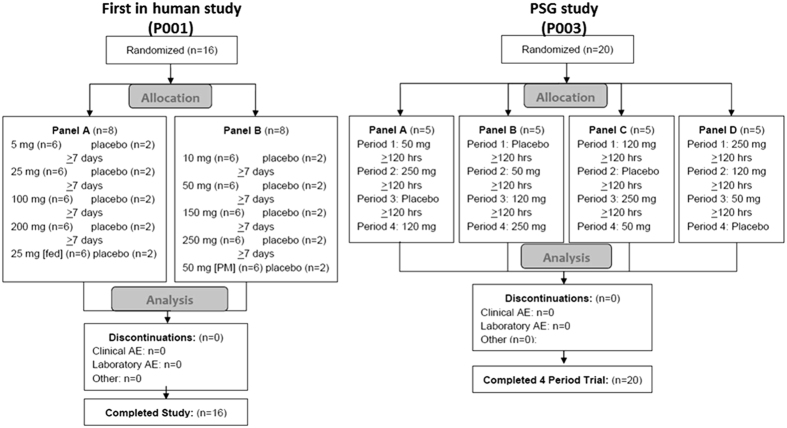
Enrolment information for Phase I studies in healthy human volunteers.

Two single-dose studies were conducted to assess the clinical safety, tolerability, pharmacokinetic (PK), and pharmacodynamic (PD) profile of MK-1064. The first-in-human study was a randomized, double-blind, placebo-controlled, alternating panel, ascending single-dose study in 16 healthy male subjects (Fig. 5). This study characterized the safety, tolerability and PK profile of MK-1064 single doses of 5–250 mg. In addition, central nervous system PD effects on arousal, somnolence and mood were assessed using the KSS and the Bond and Lader VAS. All randomized 16 male subjects (age range: 21–43 years) completed the study. The second study was a randomized, double-blind, placebo-controlled, 5-period crossover study to evaluate the effects of single oral doses of MK-1064 (50, 120, and 250 mg) administered during the evening on multiple PSG measures in 20 healthy male subjects (Fig. 5). All 20 male subjects (age range: 19–38 years) randomized in this second study completed the 4-period crossover.

MK-1064 was well tolerated in both studies, and no subjects discontinued due to adverse events (AEs). No clinically significant abnormalities were noted in routine blood and urine chemistry panels, haematology, vital signs, or ECGs, and no clinically significant abnormalities were noted in physical or neurological exams. In the ascending single-dose study, 52 AEs were considered to be related to the study drug, the most common of which was somnolence/sleepiness, drowsiness, and tiredness (15 of 16 subjects dosed with MK-1064), consistent with the intended pharmacological activity. Nine subjects dosed with MK-1064 also experienced headaches, while all other AEs were transient in duration and mild to moderate in intensity, and no suspected serious adverse reactions were observed (Supplementary Table 1). In the PSG study, 13 of the 20 subjects enrolled reported ≥1 clinical AE. Of 24 clinical AEs reported, 18 were considered to be related to study treatment, all of which were mild or moderate in intensity. One report of transient sleep paralysis following MK-1064 250 mg was noted, while the most common MK-1064-related AEs were headache (3 subjects), nightmares (2 subjects) and dizziness (1 subject) (Supplementary Table 2).

**Figure 6:**
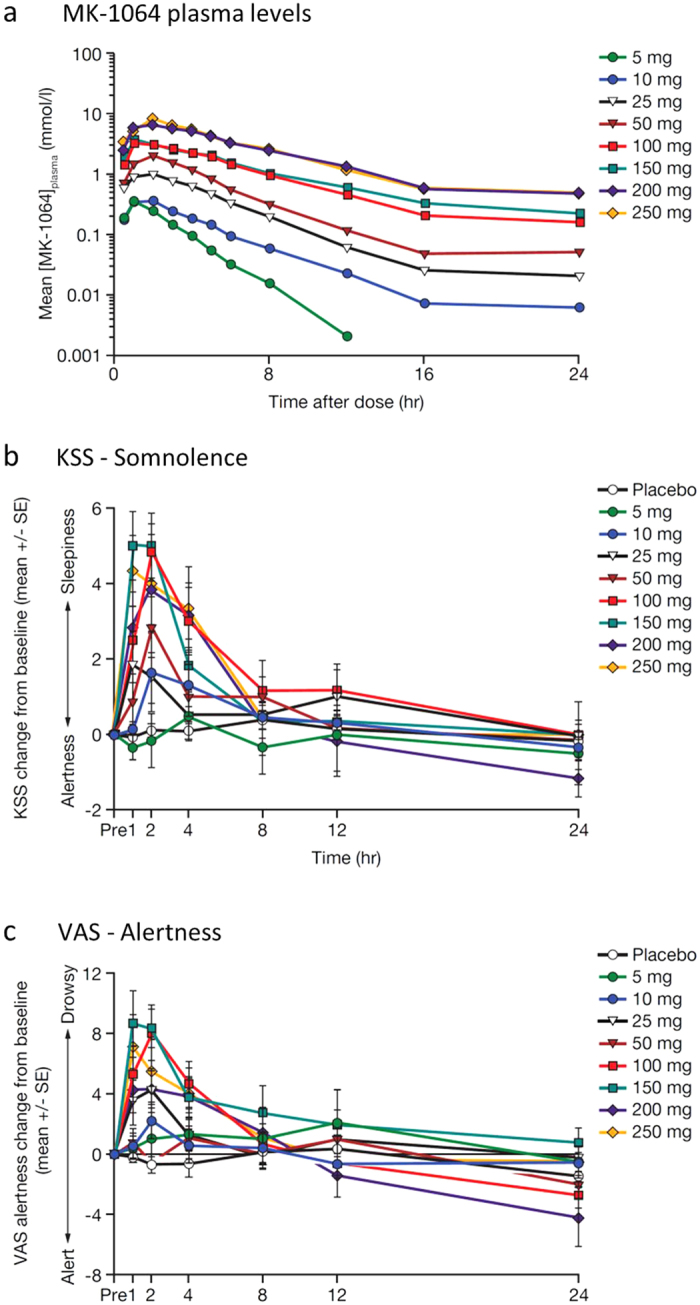
MK-1064 dose-dependently promotes somnolence and attenuates arousal in healthy human subjects. (**a**) Plasma concentration of MK-1064 over time following administration of single doses of MK-1064 5–250 mg. (**b**) Somnolence, as measured by the KSS, and (**c**) alertness, as measured by the Bond and Lader VAS, over time following administration of single doses of MK-1064 (5–250 mg) or placebo in healthy human subjects (*N* = 20).

In the ascending single-dose study, doses of MK-1064 ranging from 5 to 250 mg corresponded with dose-dependent changes in measured plasma levels (Fig. 6a). The median peak concentrations occurred within 1–2 hours (T_max_) and ranged from 0.37 to 8.28 μM (C_max_). MK-1064 plasma concentrations declined in a biphasic manner, with an alpha phase half-life of 1.6–4.0 hours and mean apparent terminal half-life of 6.8–11.1 hours (Supplementary Table 3). Dose-dependent increases in subject-reported somnolence (KSS) and drowsiness (alertness VAS) were observed following MK-1064 administration at all doses greater than 5 mg (Fig. 6b,c). The greatest effects were observed between 1 and 4 hours, and effects greatly diminished 8 hours following treatment, consistent with the time course of plasma concentrations of MK-1064.

**Figure 7:**
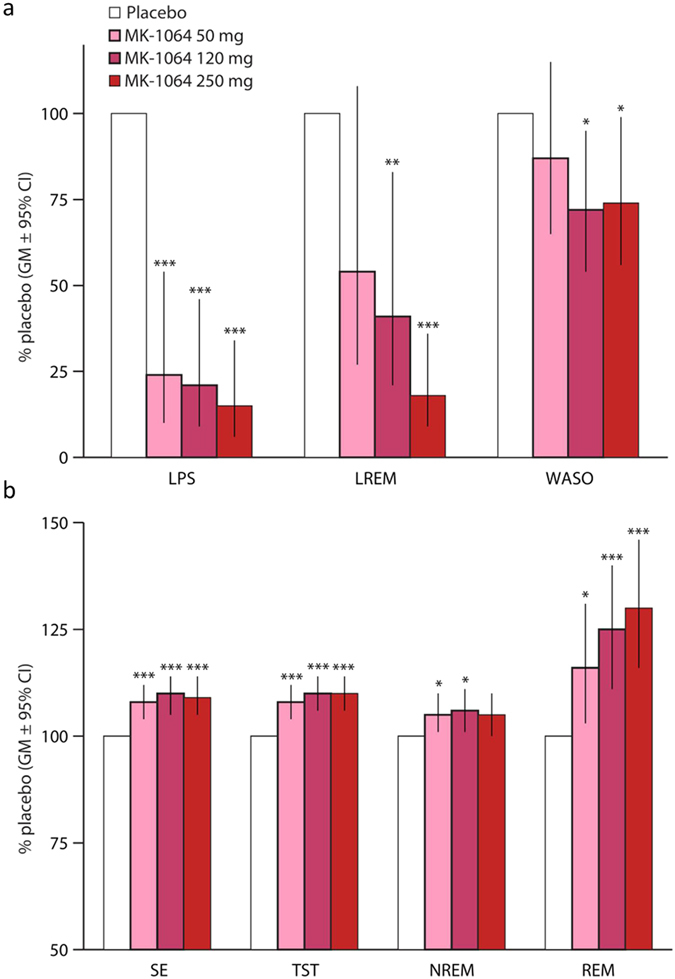
MK-1064 promotes sleep in healthy subjects. MK-1064 (50, 120 and 250 mg) or placebo were administered 1 hour prior to the normal resting phase in healthy subjects (*N* = 20), and PSG effects were monitored during the normal resting phase. Effects of MK-1064 versus placebo on time spent in (**a**) latency to persistent sleep (LPS), latency to REM (LREM), and wake after sleep onset (WASO), and (**b**) sleep efficiency (SE), total sleep time (TST), NREM, and REM are expressed as the percentage relative to the placebo condition (geometric mean [GM] ratio and 95% confidence limits [CL]; *, **, ***p < 0.05, 0.01, 0.001 respectively; mixed-effects statistical model [see Methods]).

In the PSG study, subjects underwent 8-hour PSG recording periods beginning 1 hour after nighttime administration of MK-1064 (50, 120 and 250 mg) or placebo. As evaluated as a fraction of placebo response (geometric mean ratios), MK-1064 showed significant reductions in LPS at all doses and WASO at the two highest doses (Fig. 7a). Significant increases in SE, TST and REM sleep were also observed at all doses evaluated, with increases in NREM sleep observed at the two lower doses (Fig. 7b). Reduction in the LREM with MK-1064 was statistically significant versus placebo at the two highest doses (Fig. 7a). Dose-dependence of the effects of MK-1064, while not evaluated statistically, was most pronounced with regard to LPS, LREM and REM sleep, and to a lesser extent, TST and WASO sleep (Fig. 7b). Similar to the results based on percentage differences from placebo (Fig. 7), statistically significant increases in TST, REM and NREM sleep time were observed with MK-1064 (Supplementary Fig. 2). Time spent in Stage 1 and SWS was not significantly different from placebo, while a significant increase in time spent in Stage 2 was observed with MK-1064 120 mg.

## Discussion

These studies demonstrate that MK-1064, a highly selective OX_2_R antagonist, promotes somnolence across mammalian species. Notably, increases in the mean time spent in REM and NREM sleep were observed in preclinical species as well as human subjects.

The clinical results in healthy human volunteers reported here represent the first demonstration of the sleep-promoting effects of a 2-SORA in human PSG studies. The safety profile of MK-1064 in healthy volunteers appears comparable with that seen with DORAs^10^,^34^,^35^, but further assessment in patients remains to be demonstrated. In healthy subjects, MK-1064 attenuated LPS in a dose-dependent manner and significantly increased TST and SE while reducing WASO at doses ranging from 50 to 250 mg. As such, MK-1064 exhibits characteristics consistent with those of an effective insomnia therapeutic.

Our findings also indicate that MK-1064 promotes both NREM and REM sleep, similar to sleep induced by DORAs. In rats, DORAs promote sleep that is similar to normal sleep occurring at the onset of the inactive phase, characterized by both REM and NREM sleep increases^15^. The present results are qualitatively similar to prior findings with DORAs, with little indication that the 2-SORA, MK-1064, preferentially increases NREM compared with REM sleep. DORAs also do not appear to disrupt qEEG power within sleep stages^15^,^16^,^20^. The impact of 2-SORAs on qEEG power remains to be evaluated, but given the similarity in the sleep-promoting mechanism with DORAs, clinically important differences would not be expected based on the current observations.

The current findings with a highly selective 2-SORA are consistent with preclinical genetic and pharmacological results, demonstrating that OX_2_R antagonism is sufficient to promote sleep. The role of OX_1_R in the regulation of sleep and vigilance state is clearly not as robust as that mediated by OX_2_R. Genetic depletion of OX_1_R has little effect on sleep beyond mild fragmentation^21^, and OX_1_R antagonists have little effect on sleep time when administered alone^23^,^24^,^36^. Genetic disruption of OX_2_R alone in either narcoleptic dogs or OX_2_R single knockout mice is associated with hypersomnolence associated with the narcoleptic phenotype of these animals, suggesting that reduction of OX_2_R function is sufficient for sleep^6^,^8^. The effects of the DORA, almorexant, on mean time spent in wake and sleep stages is nearly absent in OX_2_R knockout mice^22^, further supporting a predominant role of OX_2_R in sleep.

The selective localization of OX_1_R to the locus coeruleus, a brainstem structure involved in the regulation of REM sleep, suggests that DORAs may differentially impact REM sleep compared with 2-SORAs; the rationale being that REM promotion occurring via OX_1_R antagonism would be spared by 2-SORAs. However, evidence for REM promotion by 2-SORAs in previous preclinical studies has been discordant. In a study of the 2-SORA JNJ-10397049, no significant increases in the duration of REM bouts were observed, while LREM was decreased following inactive phase administration in rats; active phase treatment, however, resulted in significant increases in REM duration^23^. In contrast, Morairty *et al*. demonstrated significant and dose-dependent increases in rat REM sleep time using the 2-SORA EMPA, while no effect on REM latency was noted^24^. In another set of studies, the partial 2-SORA IPSU (6.2-fold OX_2_R/OX_1_R selectivity)^37^,^38^ showed no effect on REM sleep in mice when administered at 50 mg/kg during either the active phase^31^ or at the onset of the inactive phase^38^. While some active wake reduction was observed following active-phase dosing^31^, these results with IPSU are difficult to interpret given that the calculated OX_2_R occupancy achieved was only 48%^37^,^38^, which is well below a minimal threshold of 65% expected for OXR-antagonist–mediated efficacy^1^. MK-1064 has now been demonstrated to increase REM sleep across species including mice, rats, dogs and humans, as well as rhesus monkeys evaluated previously^29^. MK-1064 also appears to induce SWS and REM at similar OX_2_R occupancies in rats, suggesting that 2-SORAs do not differentially induce NREM relative to REM sleep, although compound-specific differences remain possible.

Orexin signaling has the potential to influence two different aspects of sleep/wake regulation; general cortical arousal and vigilance state gating. Orexin-induced arousal is thought to be mediated primarily through OX_2_R expressed on histaminergic tuberomammillary neurons, which project widely to cortical structures^7^,^24^. Antagonism of this pathway by either DORAs or 2-SORAs attenuates cortical activity and arousal, and appears to be the most salient manifestation of orexin antagonism^22^. Vigilance state gating, or the regulation of transitions between sleep/wake states, is thought to be mediated by both orexin receptors expressed in brainstem nuclei, but notably OX_1_R selectively expressed in locus coeruleus^26^,^27^. It is this second aspect of sleep/wake regulation that is expected to be differentially modulated by DORAs relative to 2-SORAs. OX_1_R has been postulated to have a role in regulating vigilance state boundaries^26^,^27^, antagonism of which could effectively diminish the threshold for sleep-state transitions. The greater OX_2_R occupancies required by MK-1064 to attenuate active wake and promote NREM and REM sleep relative to DORA-12 support the idea that additional OX_1_R antagonism acting on brain stem pathways has the capacity to contribute to efficacy. DORAs are estimated to require 65–85% occupancy to achieve efficacy^1^,^23^,^24^. MK-1064, on the other hand, attenuated active wake and increased NREM and REM sleep at substantially higher OX_2_R occupancies, the threshold for significant changes occurring between 89% and 94%. This suggests that OX_1_R antagonism inherent in the DORA mechanism may allow transitions into sleep stages to occur more readily, permitting DORAs to be more effective at lower occupancies relative to 2-SORAs. The relatively subtle effect of OX_1_R antagonism is expected to allow transitions between wake and sleep to occur more readily, but would not necessarily the total amount of time spent in one sleep stage or another. If this is true, DORAs may ultimately be found to be more effective at enabling responses to sleep debt accumulated for specific sleep stages, since the additional OX_1_R antagonism may allow these transitions to those sleep stages to occur more readily. It may also provide an explanation for discordant results by different laboratories^24^,^27^, as different animal housing conditions may predispose animal subjects to distinct sleep stages according to need. Of course, the use of distinct OXR antagonists with differing PK profiles, receptor binding kinetics and selectivity are also likely to contribute to differences in results between laboratories.

Based on FECT results, cataplexy was not observed in dogs following pharmacological antagonism of OX_2_R with MK-1064. Indeed, cataplexy was not observed in dogs at a dose of 20 mg/kg, which achieved plasma exposures of 15.3 μM that are over 500-fold exposures required for minimal sleep-promoting efficacy^29^. The lack of REM sleep increases in the hour following administration of this MK-1064 dose (see Fig. 4a) at this dose may seem surprising, but examination of the extended time course (see Supplementary Fig. 1) reveals that REM sleep was significantly increased at later time points, indicating that NREM sleep was preferentially induced over REM at earlier time points. The significance of this finding is unclear, but may suggest that acute OXR antagonism induces sleep cycling related to the timing and extent of receptor blockade, since NREM sleep typically exceeds REM sleep earlier in the normal resting period. Some OX_1_R antagonist activity may be expected at this dose particularly at earlier time points, since expected unbound drug levels at 1 hour (~T_max_) reach approximately 4.4 μM (dog-free fraction = 28.6%) that exceed the K_i_ of MK-1064 for dog OX_1_R (1.3 μM), but other off-target activity is less likely given the pan selectivity of the compound^29^. Antagonism of both OXRs, however, is not expected to be associated with positive FECT results, as no cataplectic activity of DORA-12 had been observed at doses 100-fold higher than that required for sleep-promoting effects^39^.

In summary, MK-1064 is a highly potent and selective antagonist for OX_2_Rs, promotes sleep onset and maintenance similarly across mammalian species, and is characterized by increases in both NREM and REM sleep, demonstrating that single OX_2_R antagonism is sufficient to block the arousal effects of orexin. As such, this mechanism represents a potential therapeutic strategy for the treatment of insomnia, but does not appear to offer a measurable advantage over DORAs.

## Methods

### Binding and cellular activity assays

Binding and functional blockade of OX_1_R and OX_2_R with MK-1064 relative to other 2-SORAs and the DORA, MK-6096, were assessed using membranes from Chinese hamster ovary (CHO)-K1 cells (American Type Tissue Collection, Manassas, VA) transfected to express human, mouse, rat, rabbit, dog, or rhesus monkey OX_1_R or OX_2_R receptors, as described previously^40^. The kinetics of binding to human OX_2_R was assessed by radioligand displacement on CHO-K1 cells expressing the receptor. Briefly, ^3^H-labelled ligand at K_d_ concentration was combined with 2 μg of membrane protein in 20 mM HEPES, 125 mM NaCl, 5 mM KCl buffer (pH 7.4) at room temperature. The ON rate analysis was performed by presoaking GF/B filters in 0.3% polyethyleneimine (Sigma) using a Skatron 12-well cell harvester and cold buffer at the indicated time points after addition. For OFF rate determination, binding reactions were allowed to proceed to equilibrium and terminated with 1000x [K_d_] of unlabeled antagonist and filtered as above at pre-determined time points. Ultima Gold^TM^ (PerkinElmer 6013327) scintillation cocktail was added to each filter disc and counted on a PerkinElmer TriCarb 2910 scintillation counter. Data were analyzed using GraphPad^®^ Prism v 6.04 (San Diego, CA, USA). The K_ON_ rate was determined according to the equation, K_ON_ = (K_OBS_ − K_OFF_)/[ligand].

### Animal model experiments

All studies involving animals were approved by Merck’s Institutional Animal Care and Use Committee (IACUC) and conducted in accordance with the National Research Council’s *Guide for the Care and Use of Laboratory Animals*. With the exception of rat receptor occupancy studies (as described below) experiments in mice, rats and canines did not involve anesthesia or euthanasia as part of the experimental protocol. Mouse polysomnography protocols described in Animal Procedure Statement (APS) #600775 was reviewed and approved by Merck IACUC on 31 January 2014, rat receptor occupancy described in APS #600624 was reviewed and approved on 31 July 2014, rat polysomnography described in APS #600569 was reviewed and approved on 31 May 2014, and canine polysomnography and food-elicited cataplexy test (FECT) described in APS #600645 was reviewed and approved on 31 July 2014.

### OX_2_R receptor occupancy in transgenic rats

OX_2_R occupancy in transgenic rats expressing human *Hcrtr2* cDNA via the rat enolase promoter in radioligand-binding displacement assays have been described previously^1^,^41^. Occupancy versus plasma exposure curves were first determined for MK-1064 and DORA-12 resulting from 30-minute intravenous administrations followed by euthanasia by decapitation, brain collection, tissue lysis, and measurement of radioligand displacement from 23 (DORA-12) or 59 (MK-1064) male rats. Normalized curves where the maximum occupancy value was defined as 100% relative to maximal observed plasma were constructed by fitting data to a one-site binding (hyperbola) curve (GraphPad Prism^®^ v. 6.04, San Diego, CA, USA). OX_2_R occupancy relative to changes in active wake, SWS, and REM sleep relative to vehicle were then calculated from maximum plasma values (C_max_; 1 hour) following treatment with MK-1064 or DORA-12 in satellite animals (n = 3) dosed in parallel to those used in the PSG studies, using the normalized occupancy versus plasma relation.

### Polysomnography in preclinical species

The mean time spent in sleep stages was determined in radio-telemetry–implanted mice, rats, and dogs, as described previously^1^,^14^,^40^,^42^. Continuous PSG recordings were performed following oral MK-1064 or vehicle (20% vitamin E d-alpha tocopheryl polyethylene glycol 1000 succinate [TPGS]) administration. In rodents, electrocorticogram (ECoG) and electromyogram (EMG) and locomotor activity measured via radio-telemetry were scored in 10 second epochs to define light sleep (LS; no movement, moderate EMG tone, theta with <50% delta ECoG [0.5–4 Hz]), slow wave sleep (SWS; no gross movement, reduced EMG, >50% delta ECoG) and rapid eye movement sleep (REM; no movement, little to no EMG activity, primarily theta ECoG [4–7 Hz] activity)^42^. Scored data were then grouped and compiled according to condition (vehicle or compound treatment) averaged over the indicated times (30 minutes or 2 hours) and analyzed statistically as previously described^1^,^14^,^40^,^42^ (see below). Canine sleep was scored similarly except that electrooculogram (EOG) recordings were used to define and distinguish REM sleep (high frequency EOG, no locomotor, little to no EMG activity, theta ECoG) from non-REM I (NREM I; little to no EOG activity, no movement, moderate EMG tone, <50% delta ECoG) and NREM II sleep (little to no EOG activity, no gross movement, reduced EMG, >50% delta ECoG), and analyzed as described previously^14^,^39^.

In male wild-type and *Hcrtr1* and *2* homozygous, double knockout mice (Taconic Farms, Germantown, NY), MK-1064 or vehicle was administered during the late active phase (ZT: 20:00, 4 hours before lights on). The effect of compound treatment was assessed using a counterbalanced crossover design in which all animals were alternately treated with drug and vehicle daily for 5 consecutive days with a 2-day intervening washout period. In both mice and rats, the mean time in active wake, LS, SWS and REM sleep was determined for each 30-minute interval. The level of significance for the differences between mean time spent in each stage between the vehicle- and compound-treated groups was determined by a linear mixed-effects model for repeated measures applied t-test, and mean within-subject change relative to vehicle over 2 hours following dosing was analyzed using within-subject ANOVA to determine main effects and one sample t-test to identify significant changes relative to vehicle.

In adult male Sprague-Dawley rats (600 g, Taconic Farms, Germantown, NY), MK-1064 (5, 10, or 20 mg/kg) or vehicle was administered orally in a balanced crossover design such that each subject received drug and vehicle treatments (7 consecutive days). Time in wake, light sleep, SWS and REM sleep was monitored following treatment administration during the active phase (ZT 17:00) and inactive phase (ZT 23:00; 1 hour prior to lights on). Data were analyzed using within-subject ANOVA to determine main effects and one sample t-test to compare with vehicle.

Female dogs (beagles; Marshall Farms) received MK-1064 or vehicle by oral gavage once daily at ZT 03:00 in a 5-day crossover design in which all animals received both vehicle and compound in separate arms for 5 consecutive days (1 mg/kg study) or 20 mg/kg for 12 days at ZT 04:00 in a similar crossover paradigm to match dosing times for the FECT study evaluation (see below). Mean times in active wake, NREM I, NREM II, and REM sleep were monitored continuously. Vehicle and compound treatment conditions were consolidated into 24-hour periods for each animal, and the mean time spent in each sleep stage for the 2 hours following treatment was determined and significance determined by t-test.

### Food-elicited cataplexy test (FECT)

The FECT was performed essentially as described previously^39^. After a 2-day vehicle run-in period, female dogs (*n* = 8; Marshall Farms) were treated with MK-1064 (20 mg/kg) or vehicle in a 1-day vehicle x 2-day drug crossover design. Daytime FECTs^32^,^43^,^44^ with simultaneous video and electrocorticography (ECoG), electromyography (EMG), and electrooculography (EOG) recordings were conducted 1 hour post-dose. Following release into the test room, dogs were remotely monitored while consuming 10 evenly spaced, approximately 1-inch^2^ balls of wet dog food (Pedigree^®^ chunk beef). Video and ECoG/EOG/EMG recordings were evaluated for any signs of cataplexy (e.g., collapse, EMG attenuation, sudden-onset REM sleep; see Kushida *et al*.^43^) and the time needed to consume all 10 balls of food.

### MK-1064 responses in healthy human subjects

MK-1064 was evaluated in two different studies; single oral ascending-dose first-in-human study (Merck Protocol MK-1064-001; approved by the Commissie voor Medische Ethiek – Ziekenhuis Netwerk Antwerpen, Antwerp, Belgium [#3402; 10/06/2009]; registered with the EU Clinical Trials Register [EudraCT number: 2009-010203-92; 07/07/2009] and Clinicaltrials.gov (NCT02549014; 09/11/2015); study initiation [FPI]: 07/07/2009, study completion [LPO]: 09/29/2009), and another to evaluate polysomnological responses to MK-1064 (Merck protocol MK-1064-003; approved by the Plymouth Independent Research Ethics Committee, Plymouth, U.K. [10/26/2009]; registered with the EU Clinical Trials Register [EudraCT number: 2009-014199-23; 11/26/2009] and Clinicaltrials.gov (NCT02549027; 09/11/2015); study initiation [FPI]: 11/26/2009, study completion (LPO): 04/06/2010). The authors confirm that all ongoing and related trials for this drug/intervention are registered. In both studies, all subjects provided written informed consent to participate, and were conducted in accordance with standards established by the Declaration of Helsinki, in compliance with all local and/or national regulations and directives, and approved by the Belgium and U.K. committees indicated above. Full details of the study methods are available online in the registered study materials at Clinicaltrials.gov.

### MK-1064 pharmacology, vigilance, and somnolence responses

MK-1064 safety, PK and PD were evaluated in a randomised, double-blind, placebo-controlled, alternating panel, single oral ascending-dose first-in-human study (see Fig. 5). Participants were unmedicated healthy male subjects (age 18–45 years) without a history of physical or mental illnesses or sleep abnormalities. Two panels received alternating single ascending oral doses of MK-1064 in up to 5 treatment periods (Panel A: 5, 25, 100 and 200 mg [fasting conditions], 25 mg [fed condition], or matching placebo; Panel B: 10, 50, 150 and 250 mg [all daytime administration], 50 mg [evening administration], or matching placebo). Within each period, subjects were randomized to active treatment (*N* = 6) and placebo (*N* = 2) groups. There was a minimum of 3 days between ascending doses occurring between alternating subject panels to enable safety data review; treatments within subject panels were separated by a minimum 7-day washout period. The safety and tolerability of MK-1064 were monitored throughout the study by repeated evaluations of AEs, clinical assessments (semi-recumbent and orthostatic vital signs measurements, 12-lead electrocardiograms (ECGs), physical examinations, neurologic examinations), and safety laboratory tests (chemistry, hematology, urinalysis).

During each treatment period, blood was collected for PK analysis pre-dose and at time points up to 72 hours post-dose. MK-1064 plasma concentrations were determined by reverse phase high-performance liquid chromatography and detected with tandem mass spectrometry. The lower limit of quantitation was 1 ng/mL and the linear calibration range was 1–1000 ng/mL. PK parameters were calculated with WinNonLin 5.2.1. Values below the assay limit of quantification were indicated with a zero value. PD effects on arousal and mood were assessed by the Karolinska Sleepiness Scale (KSS)^45^ and Bond and Lader Visual Analogue Scale (VAS)^46^ in exploratory endpoints.

### PSG and sleep efficacy in healthy subjects

Twenty healthy male subjects between the ages of 18 and 45 years were recruited to a double-blind, randomized, 4-period, crossover study to evaluate the effects of single doses of MK-1064. Subjects were without a history of physical or mental illnesses, had no history of sleep abnormalities, and were not regularly taking medication. Subject inclusion required a sleep score ≤6 on the Pittsburgh Sleep Quality Index at the time of screening, and qualified subjects underwent a PSG screening prior to randomization to exclude those with latent sleep disturbances. In each of the 4 treatment periods, subjects were randomized to receive single doses of MK-1064 (50, 120, or 250 mg) or matching placebo according to a balanced crossover design (see Fig. 5). Briefly, 20 subjects were randomized to 4 panels (n = 5 each). Each panel then received each of the four treatments (placebo, 50, 120, 250 mg MK-1064) in 4 different study periods where each period allocated the 4 different treatments to the 4 different subject panels. Progression through each period rotated the 4 different treatments among the subject panels such that each panel ultimately received all 4 treatments. In all 4 study periods, subjects were admitted for a pre-dose habituation night. On the following treatment night, subjects were administered a single dose of study medication in a blinded manner approximately 1 hour before their habitual bedtime. Study medication was administered following a ≥4-hour fast. Dosing in different treatment periods was separated by a washout of at least 7 days for each subject. PSG recording commenced at 1 hour post-dose and continued during undisturbed sleep for 8 hours overnight. Visual scoring of each 30-sec epoch of PSG recording to wake or one of four sleep states (S1, S2, SWS, collectively referred to as NREM, and REM) was performed according to standard and established Rechtschaffen and Kales criteria^47^. Obtained scores were then used to calculate efficacy assessment measures including latency to persistent sleep (LPS) (min), wake after sleep onset (WASO) (min), sleep efficiency (SE) (%), and total sleep time (TST) (min). LPS is the time from lights off to the first epoch of 20 consecutive epochs of non-wake stage; WASO is the time in wake stage between the onset of persistent sleep and the time of lights on; and SE is the proportion of time between lights off and lights on that was spent in sleep. In addition to efficacy parameters, latency to REM (LREM) (min), equal to the time from lights off to the first epoch of REM, and the proportion (%) of TST spent in NREM and REM were calculated. Estimation of the effects of treatment on PSG endpoints relative to placebo, including the calculation of confidence intervals and p-values were done using a mixed-effect statistical model typical for crossover studies. Specifically, logarithm of an analyzed endpoint represented the response variable, and treatment dose, treatment period, and carryover effect were fixed-effect explanatory variables. Subject effect was the random effect with the within-subject covariance assumed to be of the unstructured type. Carryover effects in this study were not statistically significant (p > 0.1) and were not included in final analysis. SAS/STAT® Version 9.3 software (SAS Institute, Cary NC) was used in the analysis.

## Additional Information

**How to cite this article**: Gotter, A. L. *et al*. Orexin 2 Receptor Antagonism is Sufficient to Promote NREM and REM Sleep from Mouse to Man. *Sci. Rep.* **6**, 27147; doi: 10.1038/srep27147 (2016).

## Supplementary Material

Supplementary Information

## Figures and Tables

**Table 1:**
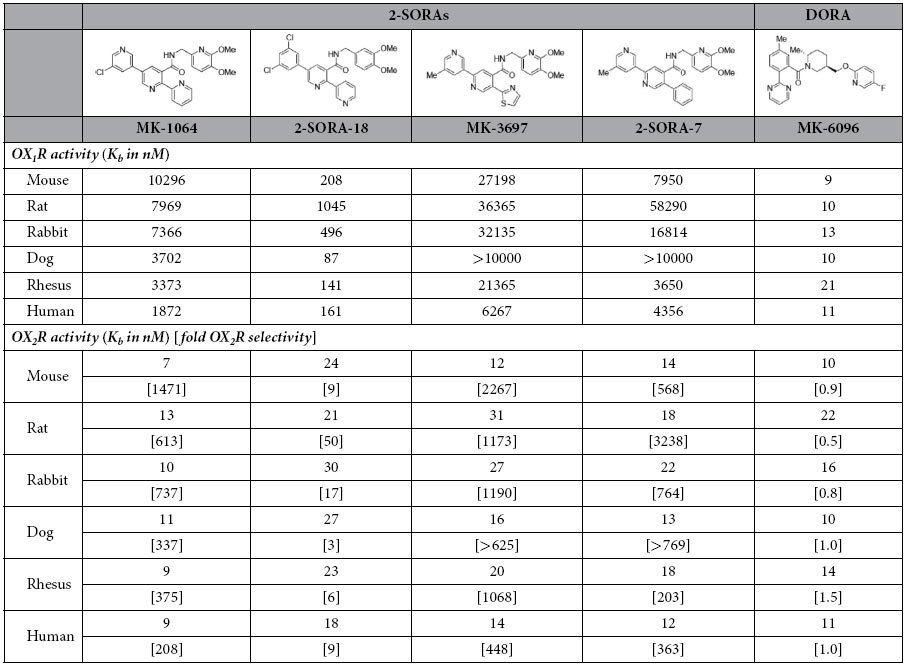
Potency of MK-1064 to inhibit OX_1_R and OX_2_R across species relative to other 2-SORAs and the DORA MK-6096.

**Table 2:** Binding of MK-1064 for OX_1_R and OX_2_R across species relative to other 2-SORAs and MK-6096 and hOX_2_R binding kinetics.

	2-SORAs				DORA
	MK-1064	2-SORA-18	MK-3697	2-SORA-7	MK-6096
*OX*_*1*_*R affinity (K*_*i*_ *in nM)*					
Mouse	1159	98	3951	1693	2.9
Rat	1069	94	2388	2613	2.5
Rabbit	2438	241	4740	4394	2.6
Dog	1300	86	3100	1800	2.7
Rhesus	815	132	2176	1336	12.5
Human	1500	100	3500	2800	2.5
*OX*_*2*_*R affinity (K*_*i*_ *in nM) [ fold OX*_*2*_*R selectivity]*					
Mouse	1.02	0.15	1.47	0.83	0.56
	[1136]	[653]	[2688]	[2040]	[5.2]
Rat	0.83	0.18	1.93	0.22	0.22
	[1288]	[522]	[1237]	[11877]	[11.4]
Rabbit	0.51	0.10	1.01	0.44	0.28
	[4780]	[2410]	[4693]	[9986]	[9.3]
Dog	0.78	0.11	1.10	0.63	0.36
	[1667]	[782]	[2818]	[2857]	[7.5]
Rhesus	0.62	0.11	1.14	0.67	0.44
	[1315]	[1200]	[1909]	[1994]	[28.4]
Human	0.52	0.10	0.97	0.47	0.31
	[2885]	[1000]	[3608]	[5957]	[8.1]
*hOX*_*2*_*R binding kinetics*					
K_d_ (nM)	0.51	ND	1.16	ND	0.34
T_1/2ON_ (min)	21.1	ND	24.3	ND	62.9
K_ON_ (min^−1^)	1.19 × 10^7^		4.69 × 10^6^		9.19 × 10^6^
T_1/2OFF_ (min)	110	ND	54.4	ND	118.3
K_OFF_ (mol^−1^min^−1^)	6.97 × 10^−3^		1.30 × 10^−2^		1.88 × 10^−2^

ND, not determined.
