# Supplementary material for: Orexin 2 Receptor Antagonism is Sufficient to Promote NREM and REM Sleep from Mouse to Man
Source: Sci Rep. 2016 Jun 3;6:27147. doi: 10.1038/srep27147 (PMC4891657; doi:10.1038/srep27147)
Supplement: Supplementary Information [file srep27147-s1.pdf]

Supplementary Information for:

Orexin 2 Receptor Antagonism is Sufficient to Promote NREM and REM Sleep from Mouse to Man

Anthony L. Gotter[1*], Mark S. Forman[2], Charles M. Harrell[1], Joanne Stevens[3], Vladimir Svetnik[4], Ka Lai Yee[5], Xiaodong Li[5,#a], Anthony J. Roecker[6], Steven V. Fox[3], Pamela L. Tannenbaum[3], Susan L. Garson[1], Inge De Lepeliere,[2] Nicole Calder,[7,#b] Laura Rosen[7,#c], Arie Struyk[2], Paul J. Coleman[6], W. Joseph Herring[7], John J. Renger,[1] Christopher J. Winrow[1]

[1]Department of Neuroscience, Merck & Co. Inc., Kenilworth, NJ, USA

[2]Department of Translational Medicine, Merck & Co. Inc., Kenilworth, NJ, USA

[3]Department of in Vivo Pharmacology, Merck & Co. Inc., Kenilworth, NJ, USA

[4]Department of Biostatistics and Research Decision Sciences, Merck & Co. Inc., Kenilworth, NJ, USA

[5]Department of Pharmacokinetics Pharmacodynamics and Drug Metabolism, Merck & Co. Inc., Kenilworth, NJ, USA

[6]Department of Medicinal Chemistry, Merck & Co. Inc., Kenilworth, NJ, USA

[7]Department of Clinical Neuroscience, Merck & Co. Inc., Kenilworth, NJ, USA

[#a]Current address: Global Biometric Sciences, Bristol-Myers Squibb, Hopewell, NJ

[#b]Current address: GlaxoSmithKline, Uxbridge, Middlesex, UK

[#c]Current address: AstraZeneca Neuroscience Innovative Medicines, Cambridge, MA

***** Corresponding author: Email: anthony_gotter@merck.com (ALG)

Supplementary Fig. 1. Time course of MK-1064 induced PSG changes in canines.

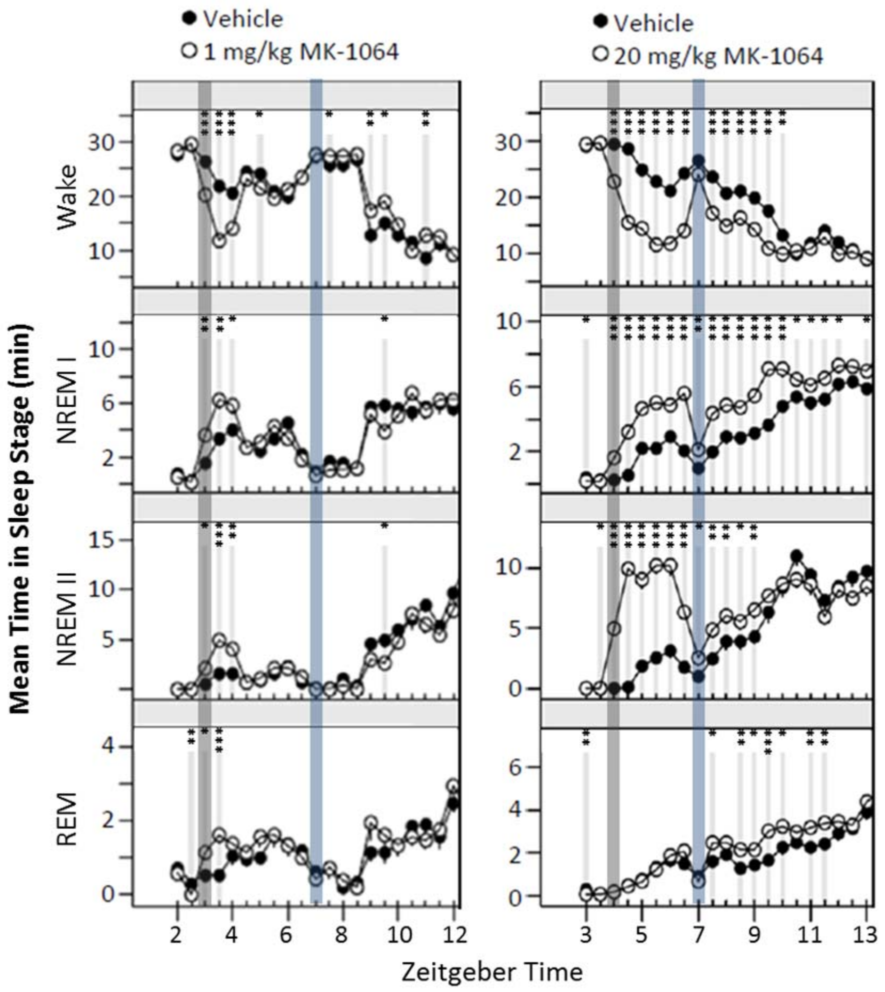

MK-1064 (open symbols) or vehicle (10 mM sodium citrate, closed symbols) was administered orally to telemetry implanted female beagles during the active phase at dosages of 1 mg/kg (n = 6, ZT 03:00, left panel) or 20 mg/kg (n = 8, ZT 04:00, right panel) (dose time indicted by vertical grey bar). Data shown represent the mean time spent in active wake, NREM I, NREM II, and REM sleep during 30-min intervals averaged from 5 consecutive days of vehicle and MK-1064 treatment occurring in a cross-over design (see Methods). Time points at which significant differences exist between vehicle and MK-1064 responses are indicated by grey vertical lines (*, **, ***: p < 0.05, 0.01, 0.001, respectively; linear mixed-effects model for repeated measures). Timing of required daily cage cleaning / water presentation is indicated by a blue vertical bar. Data presented in Figure 4a of the main article is a quantitation of the first 1 hour following administration seen in these representations. Note that while REM is not significantly increased at time points in the 3 hours following the 20 mg/kg administration, significant increases are observed at later time points.

Supplementary Fig. 2. MK-1064 increases mean TST across sleep stages in healthy subjects.

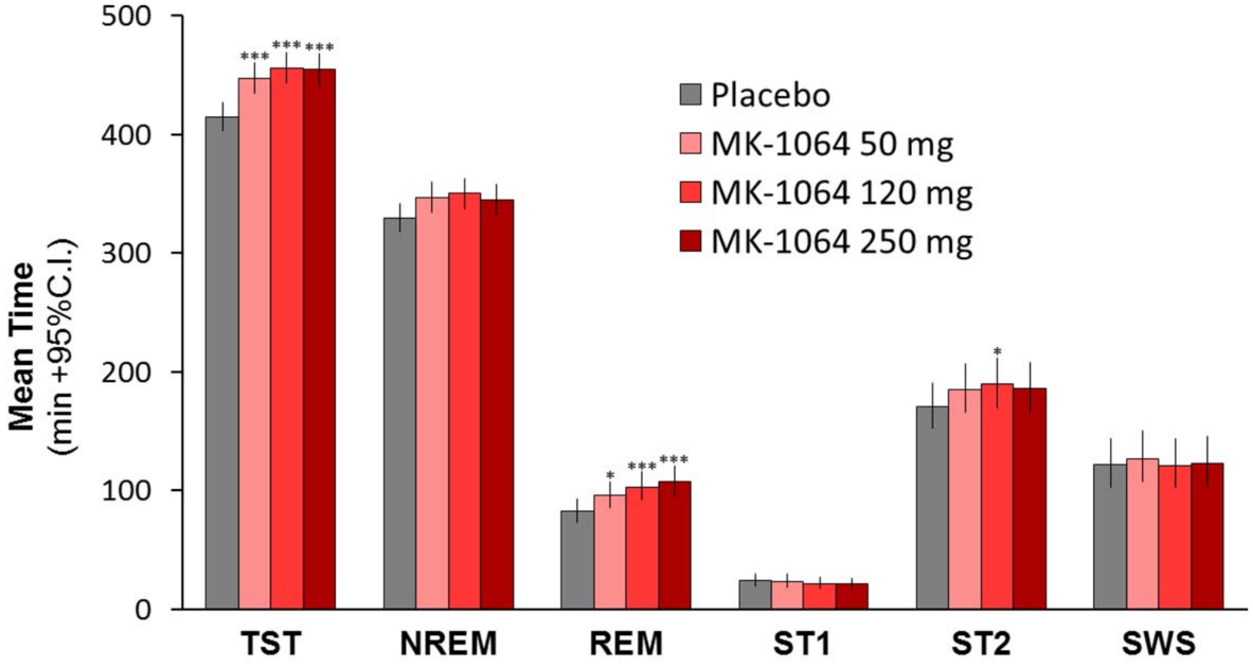

MK-1064 (50, 120, and 250 mg) or placebo was administered 1 hour prior to the normal resting phase in healthy subjects (n = 20), and polysomnographic effects were monitored during the normal resting phase. Data are presented as the geometric mean (95% +/- confidence interval, C.I.) of time spent (in min) in total sleep (TST, total sleep time) and the indicated sleep stages (NREM, non-rapid eye movement; ST1, stage 1 ST2 stage 2; SWS, slow wave sleep) in minutes. Significant differences in the geometric mean ratios relative to placebo (% placebo) are indicated: *, ***, $p < 0.05$, < 0.001.

Supplementary Table 1. Incidence of adverse events occurring during placebo and MK-1064 single-ascending dose treatment in human subjects.

	**Placebo** (*N* = 16)		**MK-1064 5 mg** (*N* = 6)		**MK-1064 10 mg** (*N* = 6)		**MK-1064 25 mg** (*N* = 6)		**MK-1064 50 mg** (*N* = 6)		**MK-1064 100 mg** (*N* = 6)		**MK-1064 200 mg** (*N* = 6)		**MK-1064 400 mg** (*N* = 6)		**MK-1064 750 mg** (*N* = 6)	
	n	(%)	*n*	(%)	*n*	(%)	*n*	(%)	*n*	(%)	*n*	(%)	*n*	(%)	*n*	(%)	*n*	(%)
Subjects with one or more adverse events	2	(12.5)	2	(33.3)	3	(50.0)	4	(66.7)	3	(50.0)	5	(83.3)	5	(83.3)	6	(100)	6	(100)
Subjects with no adverse event	14	(87.5)	4	(66.7)	3	(50.0)	2	(33.3)	3	(50.0)	1	(16.7)	1	(16.7)	0	(0.0)	0	(0.0)
**Eye disorders**	0	(0.0)	0	(0.0)	0	(0.0)	0	(0.0)	0	(0.0)	1	(16.7)	0	(0.0)	0	(0.0)	0	(0.0)
Vision blurred	0	(0.0)	0	(0.0)	0	(0.0)	0	(0.0)	0	(0.0)	1	(16.7)	0	(0.0)	0	(0.0)	0	(0.0)
**Gastrointestinal disorders**	0	(0.0)	1	(16.7)	0	(0.0)	0	(0.0)	0	(0.0)	0	(0.0)	0	(0.0)	0	(0.0)	0	(0.0)
Abdominal distension	0	(0.0)	1	(16.7)	0	(0.0)	0	(0.0)	0	(0.0)	0	(0.0)	0	(0.0)	0	(0.0)	0	(0.0)
**General disorders and administration-site conditions**	0	(0.0)	0	(0.0)	0	(0.0)	0	(0.0)	0	(0.0)	0	(0.0)	1	(16.7)	0	(0.0)	0	(0.0)
Fatigue	0	(0.0)	0	(0.0)	0	(0.0)	0	(0.0)	0	(0.0)	0	(0.0)	1	(16.7)	0	(0.0)	0	(0.0)
**Nervous system disorders**	2	(12.5)	1	(16.7)	3	(50.0)	4	(66.7)	3	(50.0)	5	(83.3)	5	(83.3)	6	(100)	6	(100)
Disturbance in attention	0	(0.0)	0	(0.0)	0	(0.0)	0	(0.0)	0	(0.0)	0	(0.0)	1	(16.7)	0	(0.0)	0	(0.0)
Dizziness postural	0	(0.0)	1	(16.7)	0	(0.0)	0	(0.0)	0	(0.0)	1	(16.7)	0	(0.0)	0	(0.0)	1	(16.7)
Headache	1	(6.3)	0	(0.0)	3	(50.0)	0	(0.0)	0	(0.0)	0	(0.0)	0	(0.0)	3	(50.0)	1	(16.7)
Somnolence	1	(6.3)	0	(0.0)	2	(33.3)	4	(66.7)	3	(50.0)	5	(83.3)	5	(83.3)	6	(100)	6	(100)
**Psychiatric disorders**	0	(0.0)	0	(0.0)	0	(0.0)	0	(0.0)	0	(0.0)	0	(0.0)	1	(16.7)	0	(0.0)	0	(0.0)
Euphoric mood	0	(0.0)	0	(0.0)	0	(0.0)	0	(0.0)	0	(0.0)	0	(0.0)	1	(16.7)	0	(0.0)	0	(0.0)
**Reproductive system and breast disorders**	0	(0.0)	0	(0.0)	0	(0.0)	0	(0.0)	0	(0.0)	0	(0.0)	0	(0.0)	0	(0.0)	1	(16.7)
Spontaneous penile erection	0	(0.0)	0	(0.0)	0	(0.0)	0	(0.0)	0	(0.0)	0	(0.0)	0	(0.0)	0	(0.0)	1	(16.7)
**Vascular disorders**	0	(0.0)	1	(16.7)	0	(0.0)	0	(0.0)	0	(0.0)	0	(0.0)	0	(0.0)	0	(0.0)	0	(0.0)
Orthostatic hypotension	0	(0.0)	1	(16.7)	0	(0.0)	0	(0.0)	0	(0.0)	0	(0.0)	0	(0.0)	0	(0.0)	0	(0.0)

Subjects are counted only once within a category regardless of the frequency of adverse events. The same subject may appear in different categories.

Supplementary Table 2. Incidence of adverse events occurring during placebo and MK-1064 administration for polysomnography analysis.

	**Placebo** (*N* = 20)		**MK-1064 50 mg** (*N* = 20)		**MK-1064 120 mg** (*N* = 20)		**MK-1064 250 mg** (*N* = 20)	
	n	(%)	*n*	(%)	*n*	(%)	*n*	(%)
Subjects with one or more adverse events	3	(15.0)	4	(20.0)	4	(20.0)	7	(35.0)
Subjects with no adverse event	17	(85.0)	16	(80.0)	16	(80.0)	13	(65.0)
**Ear and labyrinth disorders**	0	(0.0)	0	(0.0)	0	(0.0)	1	(5.0)
Tinnitus	0	(0.0)	0	(0.0)	0	(0.0)	1	(5.0)
**Eye disorders**	1	(5.0)	0	(0.0)	0	(0.0)	0	(0.0)
Blepharospasm	1	(5.0)	0	(0.0)	0	(0.0)	0	(0.0)
**Gastrointestinal disorders**	0	(0.0)	0	(0.0)	0	(0.0)	1	(5.0)
Diarrhea	0	(0.0)	0	(0.0)	0	(0.0)	1	(5.0)
**General disorders and administration-site conditions**	0	(0.0)	0	(0.0)	0	(0.0)	1	(5.0)
Chills	0	(0.0)	0	(0.0)	0	(0.0)	1	(5.0)
**Infections and infestations**	0	(0.0)	0	(0.0)	0	(0.0)	1	(5.0)
Viral upper respiratory tract infection	0	(0.0)	0	(0.0)	0	(0.0)	1	(5.0)
**Nervous system disorders**	0	(0.0)	2	(10.0)	2	(10.0)	4	(20.0)
Dizziness	0	(0.0)	1	(5.0)	0	(0.0)	1	(5.0)
Dizziness postural	0	(0.0)	0	(0.0)	1	(5.0)	0	(0.0)
Headache	0	(0.0)	1	(5.0)	0	(0.0)	2	(10.0)
Paralysis	0	(0.0)	0	(0.0)	0	(0.0)	1	(5.0)
Somnolence	0	(0.0)	0	(0.0)	1	(5.0)	0	(0.0)
**Psychiatric disorders**	0	(0.0)	1	(5.0)	2	(10.0)	1	(5.0)
Confusional state	0	(0.0)	0	(0.0)	0	(0.0)	0	(0.0)
Nightmare	0	(0.0)	1	(5.0)	2	(10.0)	1	(5.0)
**Respiratory, thoracic, and mediastinal disorders**	1	(5.0)	1	(5.0)	0	(0.0)	0	(0.0)
Epistaxis	0	(0.0)	1	(5.0)	0	(0.0)	0	(0.0)
Oropharyngeal pain	1	(5.0)	0	(0.0)	0	(0.0)	0	(0.0)
**Skin and subcutaneous tissue disorders**	1	(5.0)	0	(0.0)	0	(0.0)	0	(0.0)
Hyperhidrosis	1	(5.0)	0	(0.0)	0	(0.0)	0	(0.0)
**Vascular disorders**	0	(0.0)	0	(0.0)	1	(5.0)	0	(0.0)
Hot flash	0	(0.0)	0	(0.0)	1	(5.0)	0	(0.0)

Subjects are counted only once within a category regardless of the frequency of adverse events. The same subject may appear in different categories.

Supplementary Table 3. Pharmacokinetic parameter values for MK-1064 following administration of single oral doses to fasted healthy male subjects.

	MK-1064 dose							
	**5 mg** (*N* = 6)	**10 mg** (*N* = 6)	**25 mg** (*N* = 6)	**50 mg** (*N* = 6)	**100 mg** (*N* = 6)	**150 mg** (*N* = 6)	**200 mg** (*N* = 6)	**250 mg** (*N* = 6)
**$AUC_{0\text{-}last}$ (μM*h)** [a]	0.94 (0.25)	1.67 (0.52)	5.42 (0.71)	9.89 (3.80)	23.12 (6.23)	25.92 (6.03)	53.37 (13.78)	57.10 (21.98)
**$AUC_{0\text{-}24h}$ (μM*h)** [a]	1.95 (0.25)	1.67 (0.52)	5.22 (0.80)	9.45 (3.73)	20.93 (6.27)	23.50 (6.22)	48.93 (14.31)	51.48 (19.06)
**C_{max} (μM)** [a]	0.37 (0.007)	0.42 (0.16)	1.06 (0.22)	2.09 (0.77)	3.60 (0.85)	3.83 (1.46)	6.78 (3.52)	8.28 (2.87)
**T_{max} (h)** [b]	1.0	1.5	1.5	2.0	2.0	1.0	2.0	2.0
	(0.5–2.0)	(0.5–2.0)	(0.5–2.0)	(1.0–2.0)	(1.0–5.0)	(0.5–5.0)	(1.0–3.0)	(0.5–2.0)
**$T_{1/2}$ α (h)** [c]	1.6 (0.2)	2.5 (0.9)	2.6 (0.2)	2.5 (0.8)	3.2 (0.9)	4.0 (1.8)	3.7 (1.7)	3.6 (1.6)
**Apparent terminal $t_{1/2}$ (h)** [c]	NA	NA	NA	NA	11.1 (5.0)	6.8 (3.2)	7.5 (1.6)	8.6 (2.7)[d]

AUC, area under the concentration-time curve; C_{max}, maximum plasma concentration; T_{max}, time at which maximum plasma concentration was reached; $T_{1/2}$, half-life; ND, not determined (lack of terminal phase data precluded calculation of apparent terminal $t_{1/2}$).

[a]Mean (SD); [b]Median (minimum-maximum); [c]Harmonic mean (pseudo SD); [d]*N* = 5.
